# Modified maxillomandibular advancement for Eastern Asian patients with moderate or severe OSA: an anatomic and aerodynamic assessment of the upper airway

**DOI:** 10.3389/froh.2025.1598511

**Published:** 2025-09-11

**Authors:** Kan Li, Tianyu Zhao, Yukun Liu, Yang Cao, Xiang Li, Siyong Gao, Wei Sun, Tao Wang, Lingchan Zeng, Zhicai Feng, Guangsen Zheng

**Affiliations:** 1 Hospital of Stomatology, Guanghua School of Stomatology, Sun Yat-sen University, Guangzhou, China; 2 Guangdong Provincial Key Laboratory of Stomatology, Sun Yat-sen University, Guangzhou, China; 3 College of Stomatology, Chongqing Medical University, Chongqing, China; 4 Chongqing Key Laboratory of Oral Diseases, Chongqing, China; 5 Chongqing Municipal Key Laboratory of Oral Biomedical Engineering of Higher Education, Chongqing, China

**Keywords:** obstructive sleep apnea, skeletal class II dentomaxillofacial deformity, modified maxillomandibular advancement, three-dimensional reconstruction, computational fluid dynamics

## Abstract

**Background/purpose:**

Maxillomandibular advancement (MMA) is widely used for treating obstructive sleep apnea (OSA) patients. However, conventional MMA may not be suitable for Eastern Asian patients with moderate or severe OSA, as it can lead to unsatisfactory postoperative facial appearance. Hence, modified MMA was reported. Our study aims to evaluate the therapeutic effects of modified MMA on OSA and patient satisfaction with facial appearance. In addition, anatomic and aerodynamic changes in the upper airway were explored.

**Materials and methods:**

This retrospective study included 13 patients with moderate or severe OSA. Overnight polysomnography and the Epworth Sleepiness Scale (ESS) scores were recorded before operation and 6 months after operation to evaluate therapeutic outcomes. Spiral CT scans were performed for all patients to reconstruct 3D configurations of the bony structures and the upper airway. Computational fluid dynamics was performed to analyze aerodynamic characteristics. In addition, correlations between bone segment movements and improvement in airway parameters were examined.

**Results:**

Modified MMA achieved successful therapeutic and esthetic outcomes in all cases. The apnea–hypopnea index (36.05 ± 17.68 vs. 5.72 ± 4.76, *p* < 0.001) and the ESS (13.23 ± 8.9 vs. 6.23 ± 6.81 events/h, *p* < 0.05) decreased significantly, while the lowest oxygen saturation (76.54 ± 10.26% vs. 84.77 ± 6.02%, *p* < 0.05) improved greatly. Modified MMA significantly increased the total volume (6,716.55 ± 1,357.73 vs. 11,191.28 ± 2,563.79 mm^3^, *p* < 0.001) and the averaged cross-sectional area (117.38 ± 24.25 vs. 201.58 ± 35.76 mm^2^, *p* < 0.001) of the upper airway. After modified MMA, the pressure drop, gas velocity, and resistance in the upper airway were all significantly decreased (*p* < 0.05). Among all the maxillary and mandible sections, the strongest correlation was observed between the advanced movement of the anterior mandible segment and anatomical characteristics of the upper airway.

**Conclusion:**

Modified MMA is an excellent therapeutic method for Eastern Asian patients with skeletal class Ⅱ dentomaxillofacial deformity suffering from moderate to severe OSA; it achieves a balance between esthetic improvement and therapeutic efficacy for OSA both anatomically and aerodynamically.

## Background

Obstructive sleep apnea (OSA) is a condition characterized by repetitive episodes of apnea or hypopnea caused by pharyngeal collapse during sleep. OSA can lead to oxygen desaturation, hypercapnia, and sleep fragmentation, which contribute to cardiovascular, metabolic, and neurocognitive diseases (1). Thus, OSA is considered a potentially life-threatening disease. Multiple factors, such as obesity, excessive soft tissue in the upper airway, retrusive jaw, and dysfunction of the upper airway dilator, might contribute to the occurrence of OSA (2, 3). Currently, nasal continuous positive airway pressure (CPAP) is the first-line treatment for OSA, especially in moderate-to-severe cases. However, not all patients can tolerate lifelong CPAP use (4, 5). Since its introduction by Riley et al. in 1984, maxillomandibular advancement (MMA) has been widely used in treating OSA patients, especially in patients with retrusive maxilla and mandible (6). MMA increases the upper airway volume and reduces the upper airway collapsibility by enlarging the oral cavity, therefore alleviating OSA in patients. Over nearly 40 years of practice, MMA has been proven to be highly effective and stable for selected OSA patients (7, 8). However, in the Eastern Asian population, OSA patients more frequently present with features such as a flat nose, protrusive upper jaw, and weak chin. Thus, the conventional MMA procedure might cause adverse esthetic outcomes in these patients. Several studies reported that MMA, when modified by segmentation of the maxilla or mandible, can achieve maximal advancement while preserving a balanced facial appearance and functional dental occlusion (9–11). However, it has also been reported that a decreased oral cavity volume increases the risk of flow resistance and upper airway collapse (12, 13). We hypothesize that the airway enlargement by maxillomandibular advancement might be neutralized by the segmental setback of the anterior jaw, as the actual oral cavity volume does not change significantly.

Hence, in this study, we evaluated the clinical outcomes of modified MMA, including its therapeutic effect on OSA and patient satisfaction with facial appearance. In addition, we evaluated the airway configuration anatomically and simulated airflow changes using the computational fluid dynamics (CFD) method to gain a better understanding of the aerodynamic effects of modified MMA.

## Methods and materials

### Participants

This is a retrospective study, approved by the institutional review board of the Hospital of Stomatology, Sun Yat-sen University. Between May 2020 and March 2024, patients referred to the Hospital of Stomatology, Sun Yat-sen University, who met the following criteria were enrolled in our study:

1. Patients of either sex aged between 18 and 60 years.
2. Patients with moderate to severe OSA combined with class II skeletal dentomaxillofacial deformity, who were intolerant of other conservative treatments (e.g., CPAP, weight loss, or oral appliances).

Exclusion criteria are as follows:

1. Patients with any genetic syndromes.
2. Patients with systemic diseases contraindicating orthognathic surgery under general anesthesia.
3. Patients with uncontrolled temporomandibular disorders or periodontitis.
4. Patients with uncontrolled psychological diseases.
5. Patients who were unwilling or unable to participate in this study.

Informed consent was obtained from all participants. Approval for this research was obtained from the Institutional Research Ethics Committee (KQEC-2024-117-01).

### Orthodontic and orthognathic treatment

All patients received combined orthodontic and orthognathic treatment using a surgery-first or early approach. When necessary, limited preoperative orthodontic treatment was performed to align the dentition without further decompensation. All patients underwent modified MMA, which included conventional MMA combined with upper and lower subapical osteotomy and premolar extraction to achieve maximal mandibular and posterior maxillary advancement while avoiding postoperative bimaxillary protrusion. Genioplasty was performed when further advancement of the chin was required. Postoperative orthodontic alignment, leveling, and space management were performed to establish the final occlusion.

### Virtual surgical planning

Preoperatively, surgical simulation was performed using the Dolphin platform. Spiral CT data, digital dental casts, and 3D facial photographs were integrated into the Dolphin platform. The head orientation was set parallel to the FH plane laterally and the interpupillary line frontally. A stepwise surgical workup, including setup, cropping, cleaning, osteotomy design, landmark identification, treatment planning, presentation, and splint fabrication, was performed. An intermediate splint was designed for mandibular movement, and a final splint was designed for maxillary movement. Occlusal splints were printed by stereolithography and sterilized before surgical use.

### Assessment of patients and evaluation of sleep status

Preoperatively, the medical history and comorbidity profiles of all enrolled patients were reviewed. Physical examinations, sleep-related questionnaires, and sleep studies were conducted before and after surgery. First, physical characteristics, such as height, weight, and body mass index (BMI), were recorded. Preoperative symptoms were also carefully inquired. Then, self-evaluation of sleep quality was performed using the Epworth Sleepiness Scale (ESS) questionnaire. Finally, the apnea–hypopnea index (AHI) and lowest oxygen saturation (LSAT) were recorded using polysomnography (PSG).

### Evaluation of patients' satisfaction

Patients' satisfaction with their postoperative facial appearance was evaluated using a five-point Likert scale (1 = very dissatisfied; 5 = very satisfied).

### Assessment of the surgical movement of landmarks

**Figure 1:**
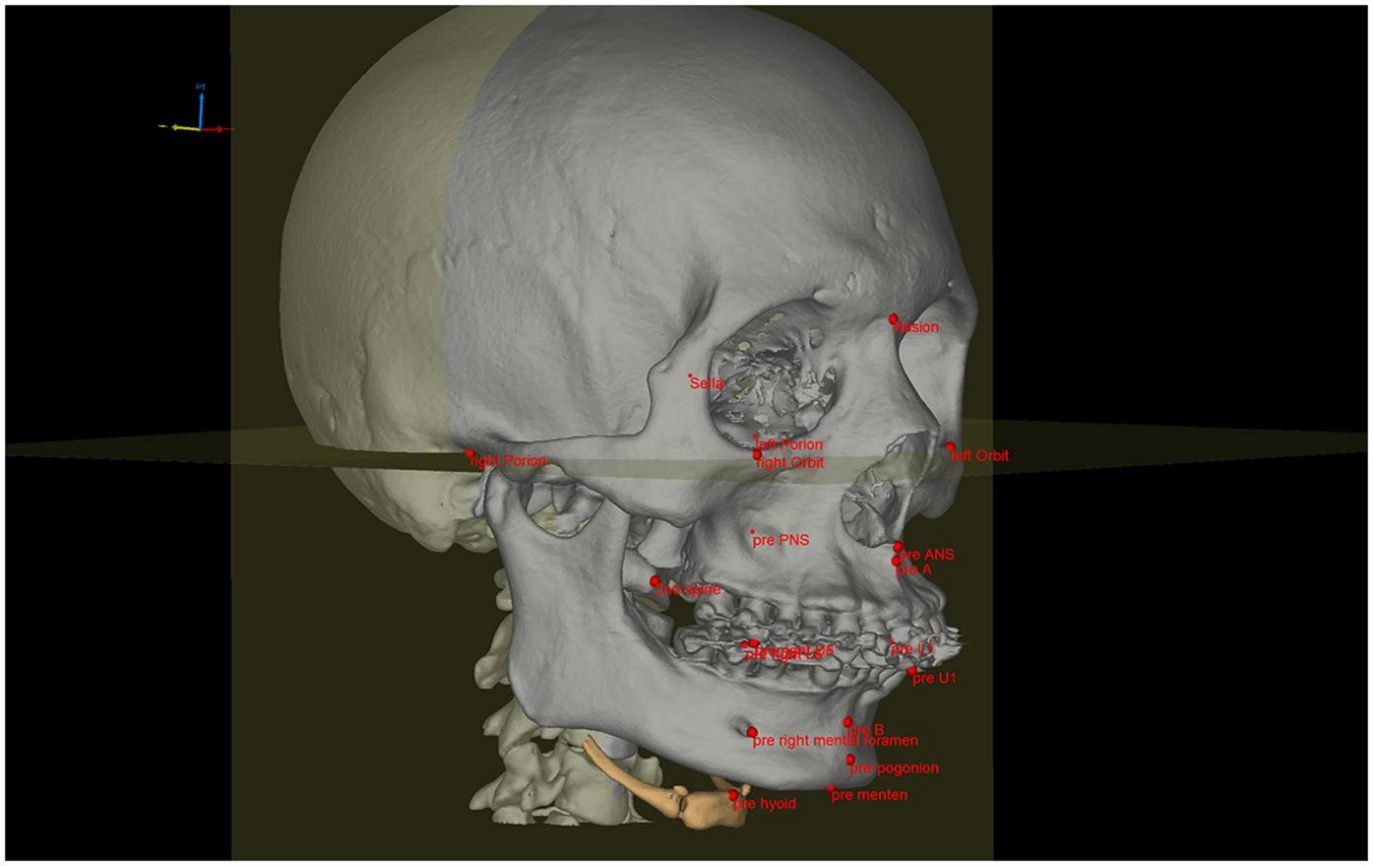
Schematic diagram of bony landmarks in modified MMA. Landmarks of the anterior maxillary segment: A point-subspinale; ANS point-anterior nasal spine; U1 point-mesioincisal angle of the upper central incisor. Landmarks of the posterior maxillary segment: U6-mesiobuccal cusp of maxillary first molar; PNS-posterior nasal spine. Landmarks of the anterior mandible segment: B-point supramental, L1-mesioincisal angle of the lower incisor. Landmarks of the posterior mandible segment: L6 point-mesiobuccal cusp of mandibular first molar; Menton point-the lowest point of mandible; Pogonion point-the most prominent point of the chin; mental foramen point-mental foramen.

After three-dimensional reconstruction of preoperative and postoperative spiral CT images, landmarks were labeled to assess the surgical movements of the jaw segments. The movement of the anterior maxillary section was measured using the A (subspinale), ANS (anterior nasal spine), and U1 (upper incisor) points, while the posterior section was marked using the U6 (maxillary first molar) and PNS (posterior nasal spine) points. The B (supramental) and L1 (lower incisor) points were regarded as landmarks of the anterior mandible segment, while the L6 (mandible first molar), Menton, Pogonion, and mental foramen points were set as landmarks of the posterior mandible segment. A schematic diagram of the bony landmarks in modified MMA is shown in Figure 1.

### Airway measurement

**Figure 2:**
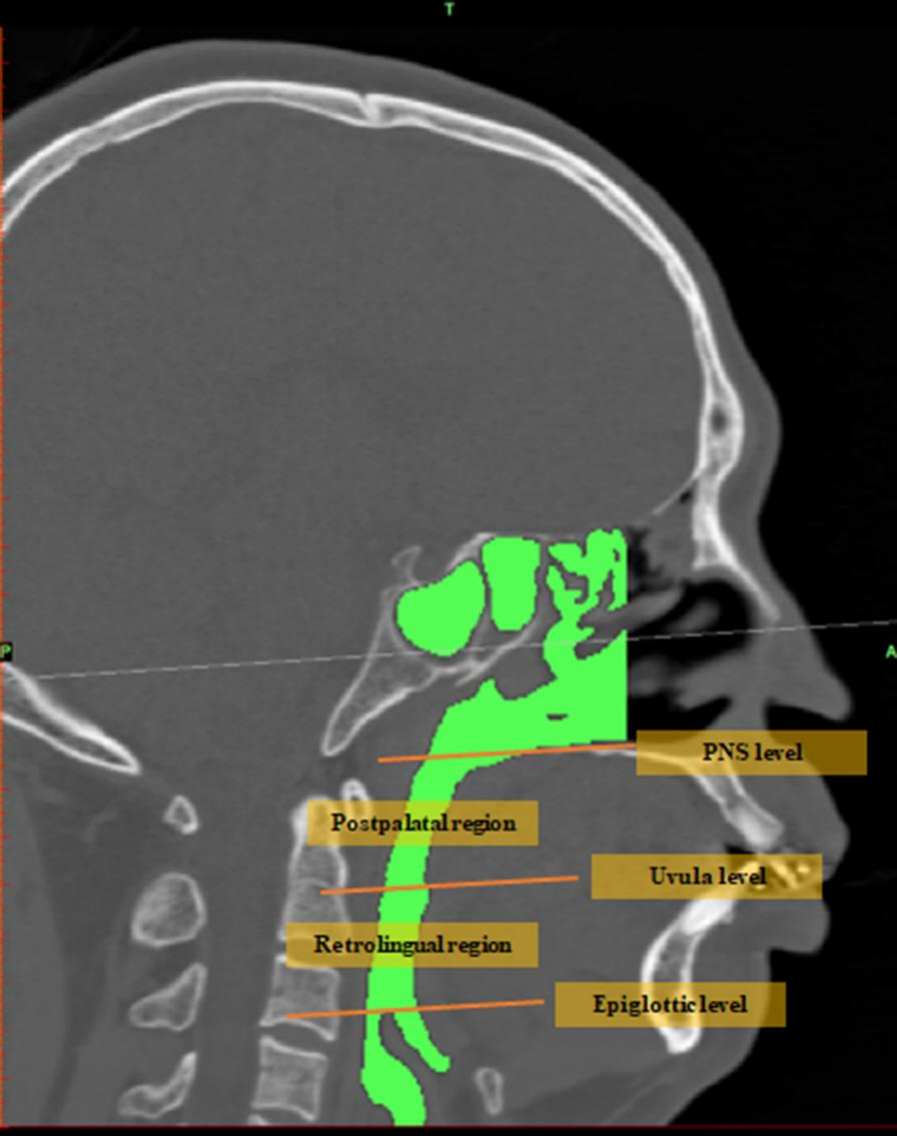
Schematic diagram of upper airway partition.

Preoperative and postoperative spiral CT data were imported into the Mimics 20.01 platform to reconstruct the jaw, airway, and hyoid. Image segmentation of these structures was performed based on the threshold values from their DICOM image series. A three-dimensional airway model was created for the region between the nostrils and the infraglottic cavity, excluding the paranasal sinuses. Then, the airway model was smoothed while preserving patient-specific characteristics. The airway was further divided into the nasal airway, postpalatal airway, and postlingual airway using three virtual planes, which were defined as parallel to the FH plane and passing through the PNS, uvula tip, and epiglottic tip, respectively. Preoperative and postoperative jaws, airways, and hyoids were matched by point-based registration of orbital and cranial landmarks. Landmarks (Figure 1) of preoperative and postoperative jaws were analyzed. Pre- and postoperative airway configuration changes were quantified by measuring total airway volume, postpalatal volume, postlingual volume, total airway surface area, postpalatal surface area, postlingual surface area, mean airway cross-sectional area, cross-sectional area at the PNS level, cross-sectional area at the uvula level, and cross-sectional area at the epiglottic level. A schematic diagram of the upper airway segmentation is shown in Figure 2.

### Computational fluid dynamics analysis

Preoperative and postoperative airway models were exported as STL files and further smoothed in the Geomagic platform. Each airway was then meshed into numerical computational cells. Laminar and turbulent CFD simulations were performed on the 3D airway models to predict the flow field in the nasal and upper airway of 13 patients before and after MMA. For numerical simulations, the inlet air pressure at the nostrils was set to ambient pressure (1 atm). A steady airflow rate of 700 mL/s, representative of adult inhalation, instead of dynamic tidal air flow, was used to calculate flow fields and pressure distributions. The same airflow rate was used for all cases. The simulation was designed to model human inspiration at rest under atmospheric pressure (1.013 × 10^5^ Pa) and atmospheric temperature (20°C). The coefficients of viscosity (1.822 × 10^5^ Pas) and density (1.205 kg/m^3^) were provided as fluid data.

### Statistical analysis

All statistical analyses were performed using GraphPad Prism 8.0 and SPSS 25.0 software. The Shapiro–Wilk (S-W) test was used to evaluate the normality of data distribution. For comparison between two groups, a two-tailed paired *t*-test was applied for the data with normally distributed differences, and the Mann–Whitney *U*-test was used for non-normally distributed ones. To analyze correlations between two groups, Pearson correlation analysis was used for normally distributed continuous data, while Spearman correlation analysis was used for ordinal variables. A *p*-value <0.05 was considered statistically significant.

## Results

### Patient characteristics

A total of 13 patients with skeletal class II dentomaxillofacial deformity were enrolled in this study. All patients presented with both mandibular retrusion and sleep apnea. Three of them also reported maxillary protrusion. Patient characteristics are summarized in Table 1. Four patients had undergone tonsillectomy, and two had tried but eventually discontinued CPAP due to intolerance. Snoring was the most frequent symptom, followed by excessive daytime sleepiness, frequent awakenings, morning headaches, and nighttime urination. One patient had systemic diseases secondary to OSA, including hypertension and stroke.

**Table 1:** Participant characteristics before surgery.

Participant characteristics before surgery, *n* (%)	
Age (years), mean ± SD (range)	27.85 ± 6.52 (20–41)
Sex, *n* (%)	
Male	9 (69.2)
Female	4 (30.8)
BMI (kg/m^2^), mean ± SD (range)	24.58 ± 2.82 (20.57–29.98)
Degree of OSA	
Mild	0 (0)
Moderate	6 (46.2)
Severe	7 (53.8)
Vertical skeletal type	
Hypodivergent	0 (0)
Normally divergent	2 (15.4)
Hyperdivergent	11 (84.6)
Preoperative symptoms	
Loud snoring	13 (100)
Awakening	7 (53.8)
Nighttime urination	1 (9.1)
Excessive daytime sleepiness	8 (72.7)
Morning headaches	6 (46.2)
Previous OSA treatment	4(30.8)

The mean BMI before surgery was 24.58 ± 2.82 kg/m^2^. Height and weight were remeasured 6 months after surgery when the patients conducted the postoperative PSG. The mean postoperative BMI was 25.10 ± 3.49 kg/m^2^. No significant differences were observed.

### Satisfaction with facial appearance

**Figure 3:**
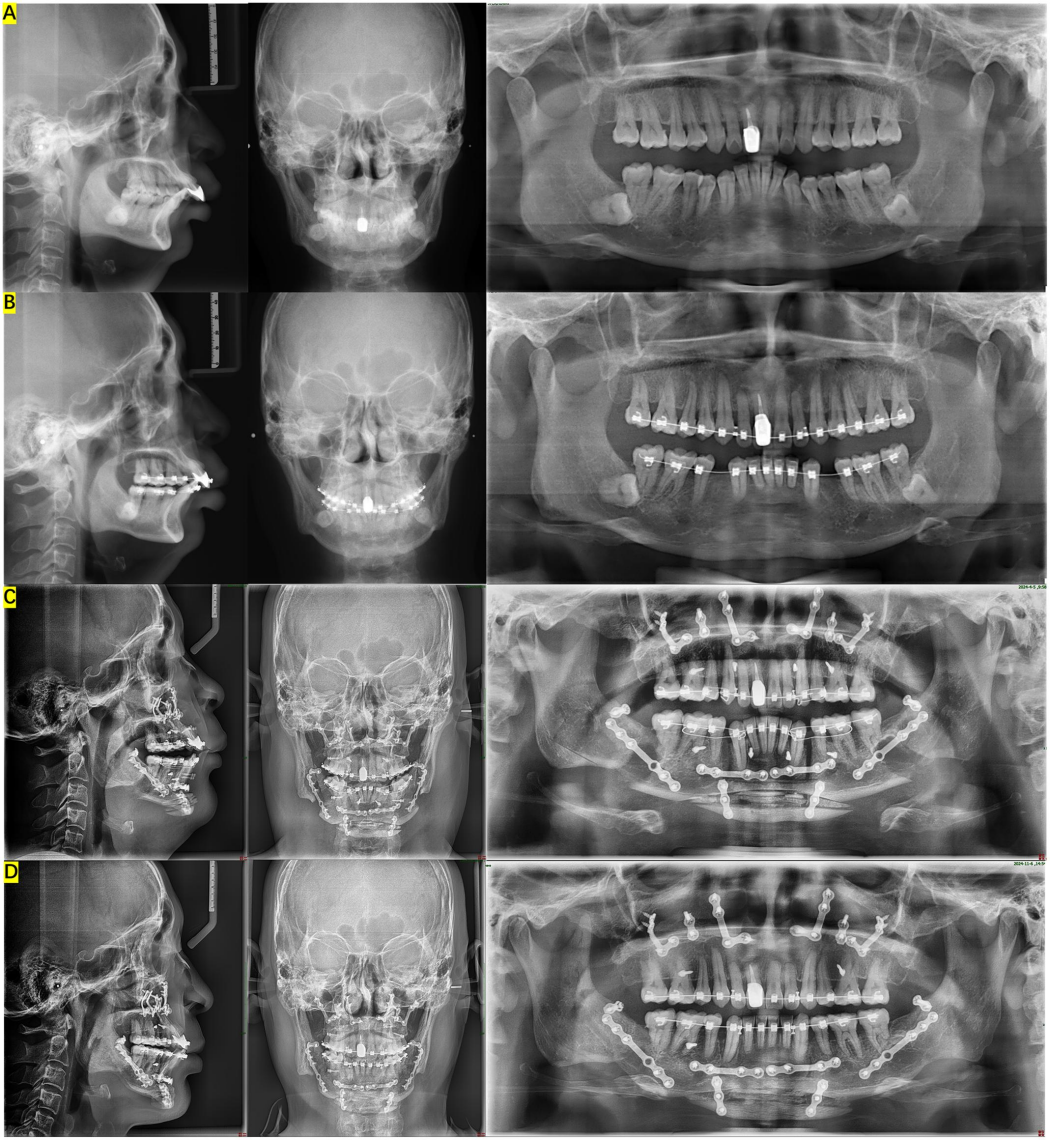
Lateral cephalometric radiograph, posteroanterior cephalometric radiograph and panoramic radiograph during orthodontic-surgical combined therapy. **(A)** Before pre-orthognathic orthodontics, **(B)** 1 week before orthognathic surgery, **(C)** 1 week after orthognathic surgery, **(D)** 6 months after orthognathic surgery.

All patients reported being “satisfied” or “very satisfied” with their postoperative facial appearance (Table 2). Radiographic records of a typical case are shown in Figure 3. None of the patients reported that their appearance had changed to bimaxillary protrusion after surgery.

**Table 2:** Likert scale for evaluating facial appearance satisfaction.

Satisfaction with facial appearance, *n* (%)	
Very satisfied	9 (69.2)
Satisfied	4 (30.8)
Neutral	0
Dissatisfied	0
Very dissatisfied	0

### Treatment effectiveness

Results of preoperative and postoperative PSG were presented in Table 3. All preoperative symptoms resolved, with only two patients complaining of slight snoring. Significant improvement was observed after modified MMA. Following the criteria of a “>50% improvement in AHI” or “post-treatment AHI < 15 events/h,” modified MMA achieved successful therapeutic outcomes in all cases. Seven patients achieved complete resolution (AHI < 5 events/h). The average reduction in the AHI was 30.32 events/h. Recovery of the LSAT during PSG was at an average of 8.23%. The ESS score decreased by an average of 8.44.

**Table 3:** Preoperative and postoperative PSG results.

Results of PSG	Preoperative		Postoperative		*p*-value
	Mean	Standard deviation	Mean	Standard deviation	
AHI (events/h)	36.05	17.68	5.72	4.76	<0.001^a^
LSAT (%)	76.54	10.26	84.77	6.02	0.018^b^
ESS	13.23	8.90	6.23	6.81	0.001^b^

a Paired sample *t*-test.

b Wilcoxon rank-sum test.

### Surgical movement of the landmarks

Surgical movement of key landmarks was also measured. The hyoid point was displaced forward by about 5.06 ± 5.09 mm and lifted up by about 4.94 ± 5.51 mm. Other detailed data are presented in Table 4.

**Table 4:** Surgical movement of important anatomical landmarks.

Surgical movement of the landmarks		Mean ± SD (range)
Forward movement (mm)	A point	−1.18 ± 1.46 (−3.41 to 1.19)
	U1 point	−1.15 ± 1.46 (−4.52 to 1.16)
	U6 point	5.79 ± 1.56 (3.17 to 8.75)
	ANS point	−2.62 ± 2.12 (−5.84 to 0.60)
	PNS point	3.97 ± 1.11 (2.4 to 5.97)
	B point	7.57 ± 4.12 (1.8 to 15.88)
	L1 point	3.4 ± 3.8 (−1.33 to 11.62)
	L6 point	9.3 ± 2.78 (6.00 to 16.55)
	Pogonion point	12.47 ± 4.77 (7.53 to 21.28)
	Menton point	13.80 ± 4.83 (8.09 to 22.62)
	Mental foramen point	10.43 ± 3.40 (7.26 to 19.38)
Degree	SNA	−1.12 ± 1.38 (−3.41 to 1.38)
	SNB	4.31 ± 2.17 (1.26 to 8.79)
	ANB	−5.13 ± 2.10 (−8.29 to −1.60)
	OP-SN	−0.37 ± 4.32 (−9.06 to 6.69)

SNA, Sella turcica-Nasion-Point A Angle; SNB, Sella turcica-Nasion-Point B Angle; ANB, Point A-Nasion-Point B Angle; OP-SN, Occlusal Plane to SN Plane.

### Morphological characteristics of the upper airway

**Figure 4:**
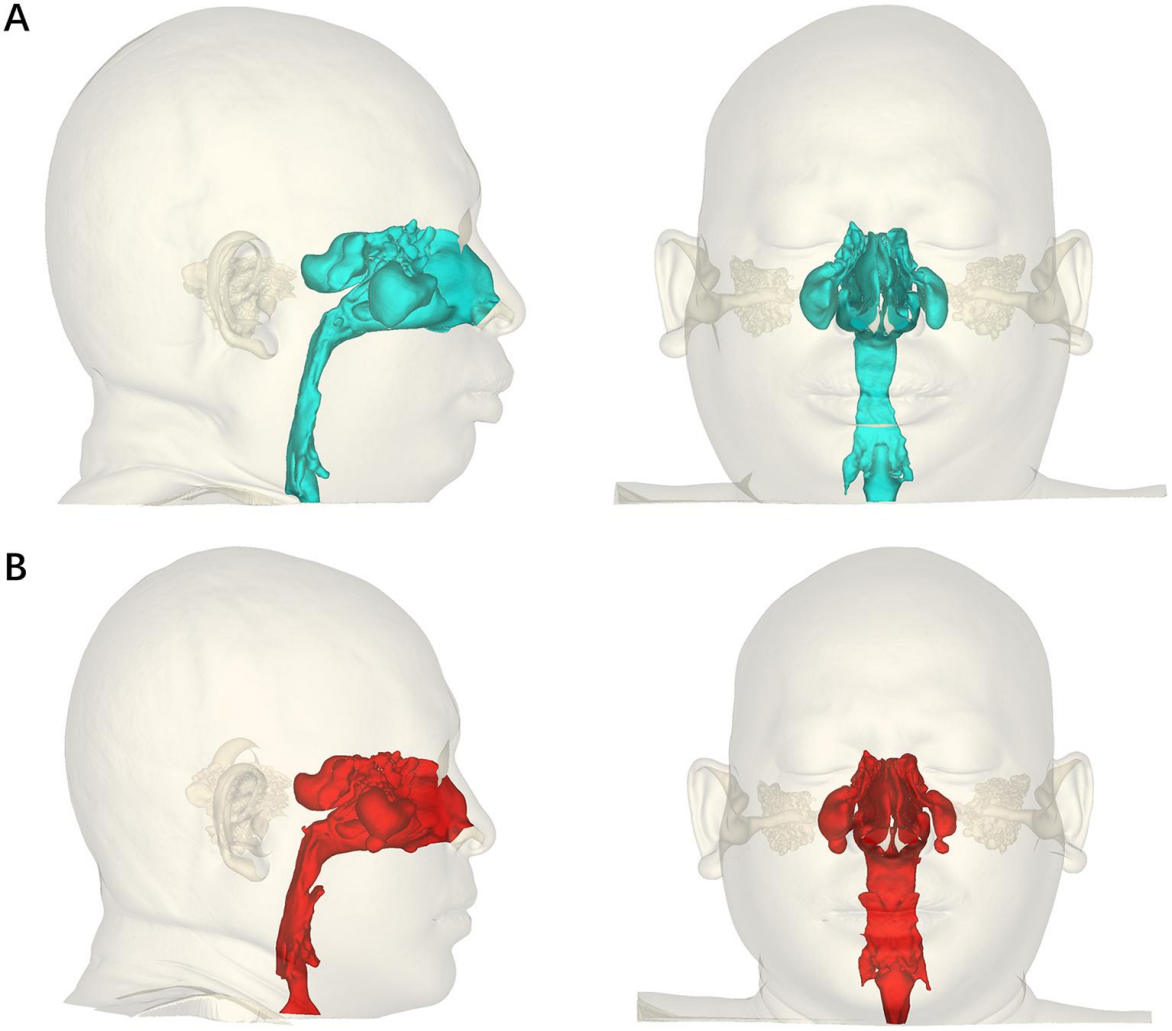
Pre-orthognathic **(A)** and post-orthognathic **(B)** three-dimensional reconstruction of the upper airway.

The 3D morphological characteristics of the upper airway are presented in Table 5, and the three-dimensional reconstruction of the upper airway is shown in Figure 4. The volume, surface area, length, and average cross-sectional area of the upper airway were measured. The postpalatal, retrolingual, and total parameters were evaluated separately. In addition, the axial area of the upper airway was evaluated at the PNS level, the uvula level, and the epiglottic level.

**Table 5:** 3D morphological characteristics of the upper airway.

Morphological characteristics of the upper airway		Preoperative		Postoperative		*p*-value
		Mean	Standard deviation	Mean	Standard deviation	
Volume (mm^3^)	Postpalatal	3,276.99	643.44	6,481.44	1,883.33	<0.001^a^
	Retrolingual	3,439.56	1,246.94	4,709.84	1,173.91	0.011^a^
	Total	6,716.55	1,357.73	11,191.28	2,563.79	<0.001^a^
Surface area (mm^2^)	Postpalatal	1,537.04	308.42	2,338.70	674.76	0.001^a^
	Retrolingual	1,393.73	425.56	1,606.31	462.22	0.072^a^
	Total	2,930.77	685.22	3,945.02	971.25	<0.001^a^
Length (mm)	Postpalatal	32.05	4.51	31.78	4.60	0.748^a^
	Retrolingual	25.92	5.91	23.48	5.65	0.033^b^
	Total	57.98	8.87	55.26	7.32	0.104^a^
Cross-sectional area (mm^2^)	PNS	284.17	84.18	432.43	147.40	0.002^a^
	Uvula	116.65	30.30	230.40	96.88	0.001^b^
	Epiglottic	180.34	89.91	237.53	95.35	0.005^a^
	Narrowest point	61.38	25.67	113.23	37.86	<0.001^a^
Mean cross-sectional area (mm^2^)	Postpalatal	103.32	21.92	202.76	48.75	<0.001^a^
	Retrolingual	136.52	47.39	206.90	54.09	0.001^a^
	Total	117.38	24.25	201.58	35.76	<0.001^a^

a Paired sample *t*-test.

b Wilcoxon rank-sum test.

Modified MMA significantly increased the volume, cross-sectional area, and average cross-sectional area of the upper airway. The postpalatal and total surface areas of the upper airway were also significantly increased after surgery. However, no significant differences were observed in the retrolingual level surface area of the upper airway between the preoperative and postoperative measurements. The length of the upper airway tended to be shortened, with no significant difference, except at the retrolingual level.

### Aerodynamic characteristics of the upper airway

**Figure 5:**
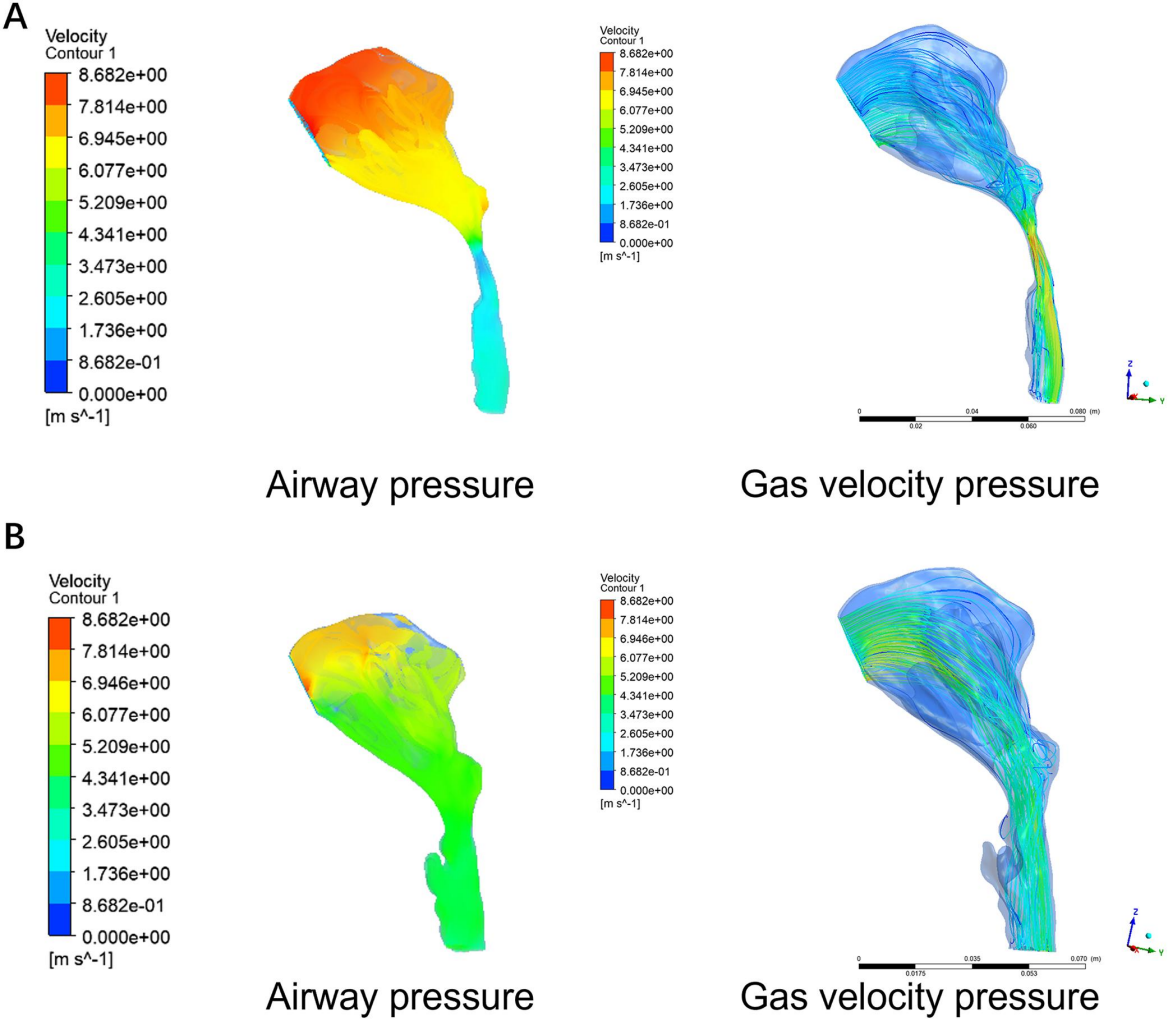
Computational Fluid Dynamics (CFD) of pre-orthognathic **(A)** and post-orthognathic **(B)** upper airway. Simulation of upper airway pressure is on the left and simulation of upper airway gas velocity is on the right.

Patient upper airway aerodynamic characteristics during inspiration are summarized in Table 6. Pressure drop, gas velocity, and resistance in the upper airway were significantly decreased after modified MMA. The simulation results of upper airway pressure and gas velocity are shown in Figure 5.

**Table 6:** Patient upper airway aerodynamic characteristics.

Aerodynamic characteristics of the upper airway		Preoperative		Postoperative		*p*-value
		Mean	Standard deviation	Mean	Standard deviation	
Pressure drop (Pa)	Total	35.54	9.22	25.79	6.53	<0.001^a^
	Nasal part	19.48	8.19	14.16	6.49	<0.001^a^
	Postpalatal part	13.7	3.21	9.22	2.07	<0.001^a^
	Retrolingual part	3.43	2.85	2.10	2.20	0.001^b^
	Postpalatal and retrolingual parts	14.45	4.54	9.70	2.85	<0.001^a^
Gas velocity (m/s)	Boundary of the nasal cavity and nasopharynx	3.41	0.78	2.72	0.71	<0.001^a^
	Soft palate	6.21	1.89	4.25	1.4	<0.001^a^
	Epiglottis	6.05	1.68	3.92	1.13	<0.001^a^
	Narrowest level	7.02	1.86	4.9	1.73	<0.001^a^
Resistance (Pa·s/m^3^)	Total	1,77,721	46,086.53	1,28,926.1	32,642.06	<0.001^a^
	Nasal part	96,130.83	39,955.22	70,776.17	32,457.93	<0.001^a^
	Postpalatal part	68,485.26	16,049.58	46,116.26	10,347.14	<0.001^a^
	Retrolingual part	17,139.98	14,271.91	10,487.14	10,997.86	0.001^b^
	Postpalatal and retrolingual parts	72,260.12	22,683	48,477.27	14,252.12	<0.001^a^

a Paired sample *t*-test.

b Wilcoxon rank-sum test.

### Correlation analysis between surgical movements of bone segments and changes in airway characteristics

**Figure 6:**
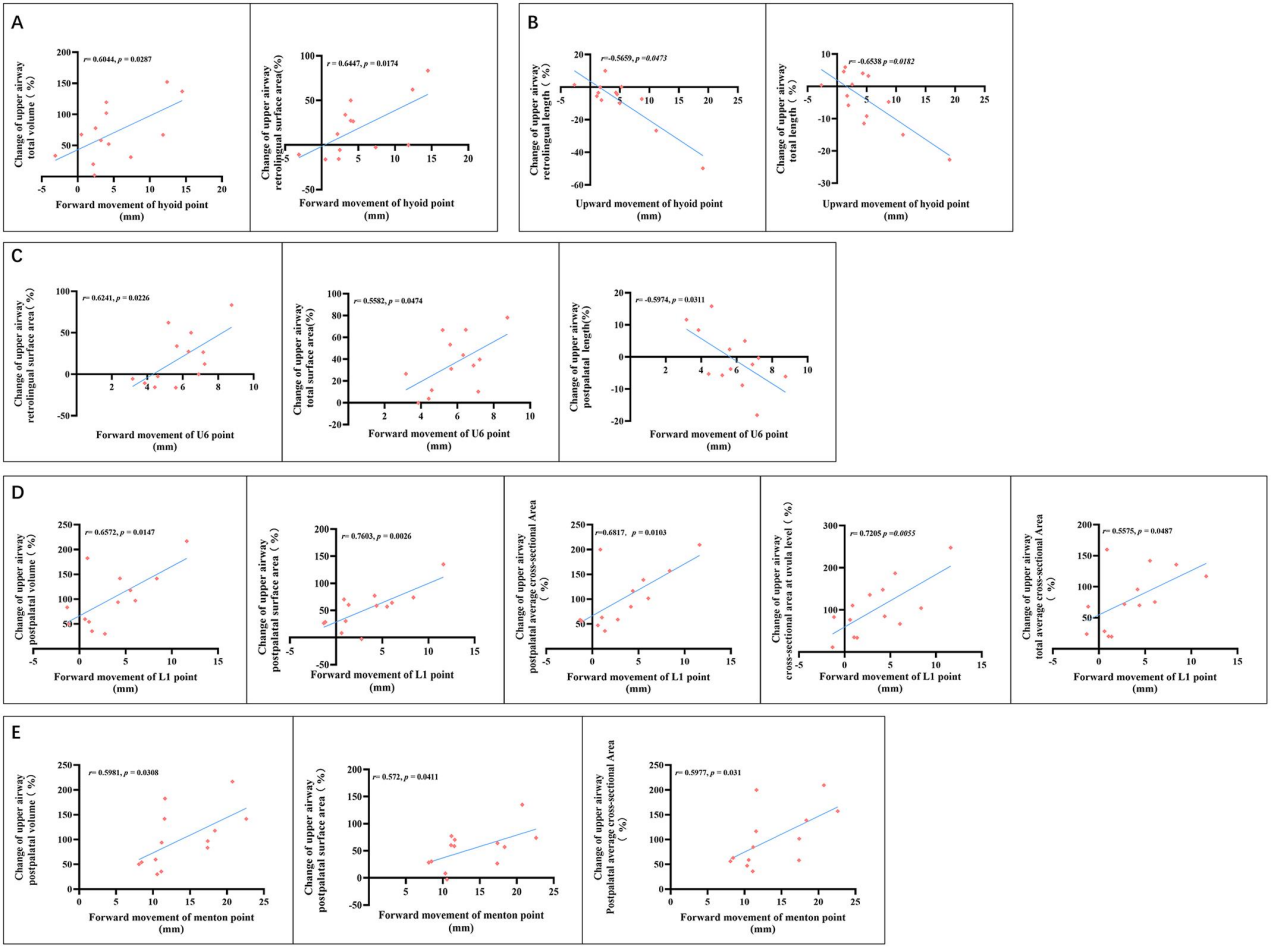
Correlation analysis between change of airway morphological characteristics and forward movement of the hyoid bone **(A)**, upward movement of the hyoid bone **(B)**, posterior maxillary segment **(C)**, anterior mandible segment **(D)**, posterior mandible segment **(E)**.

A correlation between surgical movements of bone segments and changes in airway morphologic features was observed. In detail, the change in total volume (*r* = 0.6044, *p* = 0.0287) and retrolingual surface area (*r* = 0.6447, *p* = 0.0174) exhibits a positive correlation with the degree of forward movement of the hyoid bone (Figure 6A). A negative correlation was observed between the upward movement of the hyoid bone and changes in retrolingual length (*r* = −0.5659, *p* = 0.0473) and total length (*r* = −0.6538, *p* = 0.0182) (Figure 6B). No significant correlation was found between the movement of the anterior maxillary segment and changes in any airway morphological characteristic (*p* > 0.05). Unexpectedly, aside from postpalatal length (*r* = −0.5974, *p* = 0.0311), total surface area (*r* = 0.6241, *p* = 0.0226), and retrolingual surface area (*r* = 0.5582, *p* = 0.0474), no other morphological feature showed a significant correlation with the movement of the posterior maxillary segment (Figure 6C). A strong correlation was identified between the advanced movement of the anterior mandible section and several airway characteristics, including postpalatal volume (*r* = 0.6572, *p* = 0.0147), postpalatal surface area (*r* = 0.7603, *p* = 0.0026), postpalatal average cross-sectional area (*r* = 0.6817, *p* = 0.0103), and cross-sectional area at the uvula level (*r* = 0.7205, *p* = 0.0055). Furthermore, the total average cross-sectional area demonstrated a moderate correlation with the movement of the anterior mandible section (*r* = 0.5575, *p* = 0.0487) (Figure 6D). In contrast, weaker correlations were observed between similar airway characteristics and the movement of the posterior mandible segment (Figure 6E). No correlations were found between surgical movement of bone segments and sleep-related indicators or aerodynamic parameters.

## Discussion

OSA is a very common disorder among adults. Globally, an estimated 936 million adults aged 30–69 years are affected by mild to severe obstructive sleep apnea, while 425 million within the same age range have moderate to severe obstructive sleep apnea (14). High BMI, increasing age, and male gender are regarded as risk factors for the incidence and severity of OSA (3). In our study, potential confounding variables, including sex, age, and BMI, were excluded to minimize their influence on the results.

The aim of OSA treatment is to restore adequate ventilatory function and maintain blood oxygen saturation during sleep (15). Therapeutic options can be categorized into four types: lifestyle modification, mandibular advancement devices (MADs), CPAP, and surgical interventions (4, 16). Lifestyle modifications, such as sleeping in a side-lying position and weight management, are effective for patients with mild OSA. Although CPAP remains the gold standard treatment for OSA, not all patients can tolerate lifelong CPAP use. MADs are becoming increasingly popular due to their effectiveness, convenience, and non-invasiveness; however, their use is limited by potential complications such as temporomandibular joint (TMJ) and periodontal damage. Surgical interventions are considered the last option when the non-invasive treatments fail. The aim of surgical intervention is to increase the upper airway patency at specific anatomical levels, including uvulopalatopharyngoplasty, hyoid suspension, epiglottoplasty, implantable neurostimulation devices, and orthognathic surgery (14). Since craniofacial disharmony is recognized as a predisposing risk factor for OSA, orthognathic surgery is considered the best choice to improve both ventilatory function and facial appearance (6–11).

The OSA patients in our study commonly presented with a normal or protruded maxilla combined with mandibular retraction, which is not rare in East Asia. Traditional MMA has become an effective, safe, and long-term stable treatment option for OSA (17–19). However, for these patients, the MMA is unsuitable because it may result in esthetic dissatisfaction due to postoperative bimaxillary protrusion. To avoid this problem, a balance between esthetic improvement and therapeutic efficacy for OSA could be achieved using a modified MMA technique, taking advantage of the existing edentulous spaces created after the extraction of premolars. Benito Anguita et al. reported that with traditional MMA, the mean preoperative AHI decreased significantly from 48.8 to 12.4, while ESS scores improved from 14.5 to 7.8 (17). In a meta-analysis, the mean reduction in AHI was 39.6 among Caucasians and 42.7 among other populations (18). Previous studies using modified MMA (9–11) reported successful therapeutic outcomes in all cases, with a significant decrease in AHI and marked recovery of OSA symptoms. Meanwhile, all patients reported satisfaction with their postoperative appearance. Furthermore, the surgery-first or early approach significantly shortens the lengthy process of preoperative orthodontic decompensation and prevents worsening of OSA symptoms (20), which may result from decreased oral cavity volume (12). Taken together, modified MMA is a better therapeutic option for Eastern Asian patients with skeletal class II dentomaxillofacial deformity and OSA.

Anatomically, abnormalities in craniofacial skeletal and soft tissue structures contribute to partial or complete obstruction of the upper airway in OSA patients. Neelapu et al. showed strong evidence of reduced pharyngeal airway space, inferiorly positioned hyoid bone, and increased anterior facial height in adult OSA patients compared with control subjects (2). Another study reported that upper airway collapsibility is closely associated with hyoid position, tongue volume, pharyngeal length, and waist circumference (21). A study showed that patients with a higher hyoid position responded better to MMA (19). In our study, modified MMA significantly elevated the hyoid bone and increased upper airway volume, consistent with previous reports. The increase in airway volume was closely associated with the forward movement of the hyoid bone, suggesting that OSA patients might benefit from additional hyoid suspension during modified MMA (22). Notably, movement of the hyoid bone is primarily driven by significant advancement of the mandible. Furthermore, the anterior segment of the mandible, rather than the posterior segment, demonstrates greater predictability in improving airway morphology. This finding implies that the airway enlargement achieved through maxillomandibular advancement may be counteracted if the anterior section of the mandible is set back. While the movement of the anterior maxillary section shows no significant influence on airway morphology, advancement of the posterior maxillary segment contributes to airway improvement to some extent.

Aerodynamically, airflow accelerates in narrowed regions. According to the Bernoulli effect, intraluminal pressure decreases as airflow increases. If the intra-airway pressure is lower than the external pressure, airway collapse occurs, which leads to apnea (23, 24). CFD provides an engineering modeling approach using the Navier–Stokes equation to study airway dynamics. CFD simulations showed that decreasing the airway pressure resulting from MMA decreases the breathing workload (25). It is reported that MMA increases airway volume, with a decrease in airway velocity. Significant correlations have been reported between improvements in apnea–hypopnea index values and both the increase in airway volume and the decrease in maximum airway velocity (26). Our study suggested that after removal of the airway narrow structure through modified MMA, the pressure drop, gas velocity, and resistance in the upper airway were significantly decreased.

## Conclusion and limitations

Due to the limitation of sample size, our study provides only a preliminary evaluation of the efficacy of modified MMA in treating moderate to severe OSA. Based on our existing data, modified MMA appears to be an excellent therapeutic method for the Eastern Asian population with skeletal class II dentomaxillofacial deformity suffering from moderate to severe OSA, achieving a balance between esthetic improvement and therapeutic efficacy for OSA both anatomically and aerodynamically. Further studies with larger sample sizes and comparisons with other therapeutic methods are warranted.

## Data Availability

The original contributions presented in the study are included in the article/Supplementary Material, further inquiries can be directed to the corresponding author.
